# Research on the Relationship Between Managerial Pro-Social Rule Breaking and Employees’ Workplace Deviant Behavior from the Broken Windows Effect Perspective

**DOI:** 10.3390/bs15030275

**Published:** 2025-02-26

**Authors:** Xiaoguang Liu, Wenping Liu, Safi Rubuye Deborah

**Affiliations:** School of Economics and Management, Taiyuan University of Technology, Taiyuan 030024, China; liuwenping1043@link.tyut.edu.cn (W.L.); rubuyedeborah@gmail.com (S.R.D.)

**Keywords:** managerial pro-social rule breaking, broken windows effect, organizational anomie, normative conflict, employees’ workplace deviant behavior

## Abstract

In enterprises, managers often intentionally break the rules out of altruistic motives, which is called managerial pro-social rule breaking (MPSRB). Most studies have focused on its positive consequences, while its potential dark side is neglected and lacks exploration. To bridge this gap, based on the broken windows theory, this study tries to investigate the mechanism and boundary of MPSRB’s influence on employees’ workplace deviance, introducing organizational anomie as a mediating variable and normative conflict as a moderating variable. An experiment study and a time-lagged questionnaire survey were conducted in mainland China. The results revealed the following: MPSRB had a positive impact on the perceived organizational anomie of employees; organizational anomie mediated the influence of MPSRB on employees’ workplace deviance; normative conflict moderated the influence of MPSRB on organizational anomie; normative conflict negatively moderated the indirect effect of organizational anomie. This study provides a new perspective on the mechanism and boundary of the negative consequences of MPSRB and provides practical implications for enterprises to reduce the employees’ deviance caused by MPSRB.

## 1. Introduction

Employees often face the dilemma of rigid rules conflicting with flexibility needs. For example, a customer service representative obtains an urgent order from an important client. The company rules require managerial approval to process it ahead of time, but the manager cannot deal with it promptly. So, should the representative go ahead with the order? In such cases, people may break rules for better productivity and to help colleagues or clients. This is called pro-social rule breaking (Morrison, 2006). When managers do this for altruistic reasons, it is known as managerial pro-social rule breaking (MPSRB) (Bryant et al., 2010). Scholars generally see it as beneficial leadership behavior and focus on its positive effects. From a pragmatic point of view, MPSRB helps with organizational innovation, competitive advantage, and performance (Arend, 2016; Koskela-Huotari et al., 2016; Mertens et al., 2016). Also, from the perspective of social exchange, it can promote employees’ positive attitudes and behaviors like organizational commitment, citizenship behavior, and pro-social behavior (Chen et al., 2019; Ellen et al., 2013).

However, from the perspective of social control, MPSRB may have another effect that existing studies have overlooked. The theory of containment contends that norms and laws function as external restraints to suppress deviant inclinations in individuals (Reckless, 1961). Wilson and Kelling (1982) introduced the well-known “broken windows effect” in their community security research, proposing that any environmental disorder, however minor, like breaking a window, can lead to the disintegration of external controls, resulting in increasingly serious deviant behaviors. At its core, the broken windows effect reflects rule failure, which is also frequently explored in organizational research. In an organization, the consistent compliance of organizational members with rules can contribute to maintaining the authoritative legitimacy and functional effectiveness of the organization, and symbolize its fairness, transparency, and explainability. However, some small incidents of rule breaking, even motivated by altruism, such as MPSRB, may lead employees to unconsciously believe that rules can be broken, which then triggers more and bigger deviances, but the negative effects of MPSRB are often overlooked under the cover of its “good person” aura.

Why can MPSRB act as a “broken window” in an organization, causing such harmful consequences under the radar? Because it may have a cross-norm inhibition effect, i.e., when people observe that a prohibited rule is violated with impunity, they will receive a signal that all rules are not respected, and thus reduce the tendency to comply with other rules (Keizer et al., 2008). Moreover, this signal will spread in the organization, making employees experience organizational anomie, that is, the organization is in a disordered state of rule failure. This will further motivate employees to reduce the compliance of normative goals and improve the pursuit of personal hedonic and gain goals, thus increasing deviant behavior. As a result, organizational anomie is the key mechanism of the broken windows effect in organizations. However, not all violations result in a broken windows effect, depending on how the members perceive and understand the violated rules. Keizer et al. (2011) found that the more visible, authoritative, and widely accepted a rule, the stronger the broken windows effect, which is called the cross-norm reversal effect. In contrast, when employees believe that the violated rule interferes with organizational goals and employees’ interests and there are better alternatives (Packer, 2008), in other words, when the normative conflict is higher, the more employees tend to think these rules are “bad” rules that can be violated, and the weaker the broken windows effect caused by violation of such rules.

Therefore, this paper will build a broken windows effect model of MPSRB on employees’ workplace deviance, and reveal the mediating effect of organizational anomie and the moderating effect of normative conflict. This study is helpful for organizations to take effective measures to avoid the negative consequences caused by the broken windows effect. Its theoretical contributions are as follows: Based on the perspective of social control, this paper proposes a new view that pro-social rule breaking has potentially negative effects on employees’ behaviors, which changes the focus of this kind of leadership behavior from the influence of its charitable and constructive characteristics to deviant characteristics; It builds a bridge between the broken windows theory and the theory of organizational anomie, deepens the theory about the influence of leadership behavior on employees’ perception of organizational anomie from the view of institutionalism, opens the black box of the broken windows effect of MPSRB, and expands the field in which the broken windows theory can be used for interpretation; This paper draws on the cross-norm reversal effect in the broken windows theory to analyze the moderating effect of normative conflict, which further clarifies the boundary conditions for the formation of organizational anomie under the logic of institutionalism and promotes the organizational anomie theory.

## 2. Literature Review and Hypothesis Development

### 2.1. MPSRB and Organizational Anomie

The concept of pro-social rule breaking was first proposed by Morrison (2006), who defined it as “the behavior of employees intentionally violating the formal rules or prohibitions of the organization to promote the welfare of the organization or other stakeholders”, and considered it an active altruistic behavior. Therefore, in most research, scholars regard it as a constructive organizational behavior. For example, Ellen et al. (2013) proposed that MPSRB could enhance organizational commitment or promote organizational citizenship behavior of employees. However, a few studies have pointed out that MPSRB may not only harm the interests of other groups (Dahling et al., 2012), but also cause moderate harm to employees’ work attitudes, such as reducing procedural justice and organizational identity (Bryant et al., 2010). However, there is no evidence that this kind of manager’s altruistic deviance causes employees to develop a more serious psychological risk of anomie.

The concept of anomie was first introduced by Durkheim (1893, 1951) to describe a social situation lacking norms to guide individuals in achieving goals, leading to the collapse of the social value system. Within the context of enterprises, anomie is defined from two distinct perspectives. The first perspective, rooted in the situational structure, conceptualizes anomie as the perception of an organization lacking appropriate standardization, which is consistent with Durkheim’s viewpoint (Hodson, 1999). The second perspective, drawing on social emotion, defines anomie as the dissolution of attachment to an organization, resulting in feelings of despair, helplessness, and apathy, as posited by Srole (1956). Research has identified strategic culture, competitive environment, and job design as critical factors influencing organizational anomie (Banai & Reisel, 2007; Johnson et al., 2011). In recent years, increasing attention has been accorded to the impact of leadership behavior on organizational anomie, which can be elucidated through two primary lenses. The first, grounded in the resource conservation perspective, suggests that leadership behaviors influence organizational anomie by depleting work and psychological resources (Akhtar & Shaukat, 2016; Jiang et al., 2019), aligning with the social-emotional definition and representing the current mainstream view. The second perspective, based on institutional logic, posits that leadership behaviors that erode the organizational system’s authority lead to anomie through the creation of a deficient normative work environment (Huang & Chen, 2017; Usman et al., 2020), consistent with the situational structure definition.

Although the MPSRB is motivated by altruism, it is still a kind of deviance that may decrease the subjective authority of organizational norms. So it can be explained by the logic of institutionalism. Keizer et al. (2008) found that when people see graffiti everywhere on a wall with an “anti-graffiti” sign, it significantly increases their tendency to litter. This is called the cross-norm inhibition effect, in other words, when people see that one norm is not being followed, it can inhibit an individual’s tendency to follow other unrelated norms (Lindenberg et al., 2021). MPSRB can likewise give rise to this effect. When a manager breaks a “bad” rule without facing correction or punishment, it conveys a heuristic cue to subordinates, making them perceive that rules are of little importance and that other “good” rules may also be violated by others at will. Consequently, employees come to believe that the authority of the organization’s rule system is undermined, the binding force of rules is contested, and the enforceability of rules is compromised (Offstein & Chory, 2017). They may also hold the view that management lacks either the capacity or the inclination to uphold organizational norms, thereby diminishing their sense of awe for rules. Moreover, the broken windows theory also underscores that minor violations can set off imitation effects. When employees emulate managers’ pro-social rule breaking, they may determine whether to break a rule based on their personal values or situational requirements. Leveraging the guise of pro-social, they might engage in self-serving rule breakings while claiming to be “helping others” or “doing good for the organization”. In this way, they extend pro-social rule breaking to non-prosocial contexts and rationalize it. In such an organizational context lacking clear work norms, employees will face higher role ambiguity and role conflict, and will easily have a sense of loss of control over work (Usman et al., 2020), increasing uncertainty and work alienation caused by a situation of normlessness, and ultimately increase the anomie. Thus, we propose the following hypothesis:

**Hypothesis** **1:** *MPSRB has a positive effect on the organizational anomie.*

### 2.2. The Mediating Effect of Organizational Anomie

A study of the broken windows theory found that a small violation not only makes residents feel that society is out of control but also causes more deviance in others (Wilson & Kelling, 1982). This study extends the broken windows effect to organizational management, investigating how MPSRB leads to more workplace deviance through perceived organizational anomie. Workplace deviance is a kind of negative behavior in which employees intentionally violate organizational rules and harm the interests of the organization or employees, including organizational deviance and interpersonal deviance (Bennett & Robinson, 2000). Research shows that in addition to job characteristics (Liu et al., 2021), personality traits (Aminah et al., 2017), and leadership styles (Wu et al., 2020), cognitive factors such as organizational commitment, perceived organizational inequality, organizational values, norms, and other organizational cultural factors are the more direct antecedents of employees’ deviance (Abbasi et al., 2020; Aryati et al., 2018; Di Stefano et al., 2019).

Organizational anomie, as a serious negative perception of organizational norms, can also cause workplace deviance. This can be explained by the goal-framing theory of Lindenberg and Steg (2007). This theory proposes that three overarching goals influence individual behavior: hedonic goals aim to feel good right now and avoid unpleasurable effort or discomfort, gain goals aim to preserve and improve available resources, and normative goals focus on acting appropriately (Lindenberg et al., 2021). Among them, hedonic goals and gain goals pay more attention to the actual needs of individuals, while normative goals pay more attention to the collective needs (Lindenberg & Steg, 2007). Only when the norms and institutions can form effective constraints on collective action, individuals perceive that their compliance with norms is valuable, so they will pursue normative goals more and abandon hedonic and gain goals (do Canto et al., 2023). However, when an individual observes that the organizational norms are violated and not corrected in time, it weakens the constraints of organizational rules on individual behavior and puts the organization in a situation of normlessness. He will disrespect not only the violated norm but also other related norms, lowering the salience of the individual’s own normative goal (Lindenberg et al., 2021). Therefore, consistent with the broken windows effect, in the organizational context, the MPSRB will also destroy the normality of the work environment and increase the organizational anomie (Tsahuridu, 2006). Employees will further weaken the pursuit of organizational normative goals and make them more inclined to violate organizational norms to achieve hedonic and gain goals (Bergquist et al., 2023), increasing organizational deviance. Thus, we propose the following hypothesis:

**Hypothesis** **2:** *The organizational anomie mediates the influence of MPSRB on employees’ workplace deviance.*

### 2.3. The Moderating Effect of Normative Conflict

Numerous studies have focused on exploring the boundary of the influence of leadership behavior on organizational anomie. However, the influences explored have solely emanated from the logic of resource conservation. For example, scholars have explored the moderating effects of psychological capital (Vanderstukken & Caniëls, 2021), willpower and persistence (Sarwar et al., 2022), leader–member exchange (Yu et al., 2021), and other factors, while the boundaries of this effect under the logic of institutionalism remain unclear. Keizer et al. found that people are more likely to litter when a conspicuous “no graffiti” sign is on a graffitied wall than when there is no such sign (Keizer et al., 2011). This cross-norm reversal effect further deepens the broken windows effect and clarifies its boundaries. That is, when an individual sees a violated norm as more prominent and explicitly prohibited, they feel the norm’s ineffectiveness, lower their compliance with normative goals, and achieve hedonic and gain goals by violating other unrelated norms. Consequently, whether MPSRB can cause more organizational anomie among employees depends on whether the violated rules are prominent, widely accepted, and respected. More broadly, it depends on whether the overall system of organizational norms is prominent, widely accepted, and respected.

Packer proposed the concept of normative conflict to bridge the contradictory influence of organizational identity on norm compliance (Packer, 2008), which refers to the conflict between the perceived organization’s current norms and the better alternative behavioral standards (norms). This alternative norm may stem from an individual’s identity in other areas, such as their beliefs and values (Dahling & Gutworth, 2017; Packer, 2008). Most current studies on normative conflict aim to bridge the two different effects of organizational identity on pro-organizational deviance, they propose that in a high normative conflict context, those with high organizational identity are more inclined to pro-organizational deviance, and vice versa. These studies are about dissent (Packer et al., 2014), whistleblowing (Anvari et al., 2019), and constructive deviance (Dahling & Gutworth, 2017). In addition, the moderating effects of normative conflict are widespread in other mechanisms related to altruistic deviance. For example, the psychological reaction to pro-customer deviance (Kim & Zhan, 2023), and moral judgment of others’ altruistic disclosure (Rios & Ingraffia, 2016). Therefore, this paper argues that normative conflict also moderates the effect of MPSRB on organizational anomie.

Employees with a high perception of normative conflict will perceive many of the organization’s current formal rules as outdated and in conflict with alternative norms based on the organizational goals, national culture, and prevailing values (Packer, 2008). This will cause employees to feel uncomfortable and try to find relief. In such situations, employees not only tend to make their behaviors more in line with the alternative norms, but also make positive evaluations of other people’s altruistic deviance in line with the alternative norms (Rios & Ingraffia, 2016). They deem the overall organizational system outdated, wrong, in need of change, and possibly even counter-productive (Dahling & Gutworth, 2017), constructing new standards of behaviors, and replacing organizational rules with better norms. Consequently, they see the rules violated by MPSRB as unimportant, unacceptable, lacking deterrence and authority, and can be violated. Therefore, this behavior will not be perceived as a violation of norms, and will not destroy the normalization of the organization, but rather is consistent with those alternative norms, resulting in lower organizational anomie.

Conversely, those with a low perception of normative conflict regard current norms as acceptable and prioritize strict adherence (Zhang et al., 2022). When they observe that these norms are violated by others without being held accountable, they will think that even such good norms can be violated arbitrarily, let alone other norms. As a result, their perception of the effectiveness and deterrence of the organization’s norms will be significantly reduced (McDonald et al., 2013). In such situations, not only do they perceive that complying with current norms will not achieve the desired results, but the tendency to violate other norms will also increase (Lindenberg et al., 2021), which will lead to a work environment lacking effective norms. In their view, even motivated by pro-social motives, managers’ violation of norms will make the current norms constantly changing and chaotic, and even destroy the established value system. Moreover, as an important source of information in the workplace, managers’ norm violations have contagion effects and will be imitated by multiple group members, which will promote an environment of dysfunctional organizational norms and exacerbate organizational anomie. Thus, we propose the following hypothesis:

**Hypothesis** **3:** *The positive effect of MPSRB on organizational anomie is moderated by normative conflict, the lower the normative conflict, the stronger the effect will be.*

The relationship revealed by Hypothesis 2 and Hypothesis 3 jointly implies a moderated mediating effect, that is, the normative conflict plays a moderating role in the indirect effect of MPSRB on employees’ workplace deviance through organizational anomie. For employees who perceive low normative conflict, observed MPSRB implying norm violation can contrast with the inviolability of the norm, which facilitates the collapse of organizational normative perception and enhance the organizational anomie. This enables them to lower their pursuit of normative goals, and achieve hedonic and gain goals to satisfy personal interests by deviance. Thus, we propose the following hypothesis:

**Hypothesis** **4:** *The positive mediating effect of organizational anomie is moderated by normative conflict, the lower the normative conflict, the stronger the indirect effect will be.*

### 2.4. Control Variable

Although the control variable is not the focal point of the study, it is important for the theoretical model (Becker et al., 2016). According to the studies of Semmer et al. (2010) and Lian et al. (2022), we added the managerial counter-productive behavior (MCWB) as a control variable in the research model.

Counterproductive work behavior (CWB) encompasses a range of norm-violating organizational behaviors (Vardi & Weitz, 2004) and shares similarities with (M)PSRB as both involve intentional deviations from established organizational rules; hence, they may be positively related (Galperin, 2005). However, traditional definitions of CWB primarily focus on the behavior itself, neglecting consequences and motives (Gruys & Sackett, 2003). Hence, it is too narrow to understand CWB as always self-interested and destructive (Morrison, 2006). Although CWB can sometimes be harmful to an organization or some individuals, it may be constructive for others (Cropanzano et al., 2017). Therefore, the measurement of MPSRB can explain considerable variations in managers’ CWB. The broken windows effect of this norm violation has been extensively studied in the previous literature, without distinguishing whether the motive is altruistic or not. Thus, the variations in managers’ CWB can be considerably explained by the measurement of MPSRB, and both can have the broken windows effect and cause employees’ workplace deviant behavior. All these satisfy the requirement of being a good control variable (Becker et al., 2016). Thus, we proposed the hypothesis as follows.

**Hypothesis** **5:** *The indirect effect of MPSRB on employees’ workplace deviance via organizational anomie remains significant after controlling for managerial counterproductive work behavior.*

**Figure 1:**
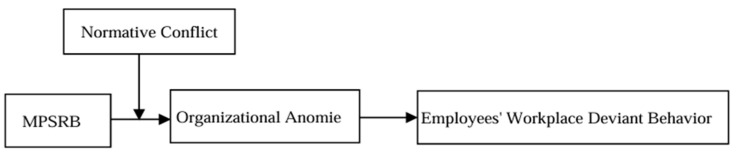
Theoretical Model.

In summary, the theoretical model of this study is depicted in Figure 1.

## 3. Research Overview

Two studies were conducted to test our hypotheses. In Study 1, to reduce the interference of measurement error and other uncontrollable factors on causal relationships, we conducted an experiment to examine the effect of MPSRB and its interaction with normative conflict on organizational anomie (i.e., Hypotheses 1 and 3). As manipulating mediating variables like organizational anomie is experimentally difficult to accomplish (Ge, 2023), organizational anomie’s influence on deviant behavior was absent in Study 1. To address this, in Study 2, we carried out a multi-wave questionnaire to test all hypotheses, especially those not covered in Study 1. These two studies supported the broken windows effect of MPSRB and enhanced the validity of our research.

## 4. Study 1

### 4.1. Participants

G*Power 3.1 software was used to determine the total sample size necessary for this study (Faul et al., 2007). The results showed that the experiment required a total sample size of at least 128 (alpha = 0.05, power = 0.8, f = 0.25). We invited 250 MBA students from a university in northern China to complete the experiment, they had certain work experience and could effectively perceive the experiment situation. Finally, 182 participants completed the experiment, of which 17 were excluded because they had failed at least one attention-checking item (see also Abbey & Meloy, 2017). There was no obvious difference between the excluded and retained samples after comparison. Thus, we obtained a valid sample size of 165 (average age = 31.1 years, SD = 0.720, 51.5% were male, 56.4% participants had been working in their organizations for 1–3 years, and 70.9% were frontline employees). At the end of the experiment, we gave each participant 15 RMB (approximately 2.07 dollars) as a cash reward.

### 4.2. Procedure and Manipulation

The research was carried out in a computer classroom at the university. Before the start of the experiment, we made sure that the participants knew nothing about our hypotheses and promised that the experiment would be completely confidential and used only for academic research, the content of the study involved organizational situations and workplace behavior. Participants were asked to read and fill out the informed consent for the experiment and report their demographic information. We designed a 2 (MPSRB: high vs. low) × 2 (normative conflict: high vs. low) experiment and participants were randomly assigned to one of four experimental conditions. All the experimental materials were displayed on the computer screen, and the participants followed instructions to complete the experiment.

At the start of the experiment, an organizational scenario adapted from Morrison’s work (Morrison, 2006) was described. Participants were asked to imagine working in a customer service department and observed their supervisor, Andy, facing the dilemma of breaking an organizational rule to handle an urgent, company-beneficial order. Scenario details were located in Appendix A. MPSRB and normative conflict were manipulated in a random order to prevent the sequence of materials from affecting the participants. After that, the participants completed the measurement of organizational anomie. Finally, they answered the questions of manipulation check and attention check.

The manipulation of MPSRB is adapted from the script of the PSRB used by Zhu et al. (2023). In the condition of high MPSRB, participants received the message “Since missing this order may bring losses to both the company and the customer, Andy decides to deal with the customer’s order without the CCO’s approval”. In the condition of low MPSRB, they received the message “Considering the company’s special rules on rush orders and the CCO’s failure to respond to requirements, Andy decides not to deal with the customer’s order without the CCO’s approval”.

The manipulation of normative conflict is mainly based on Packer’s (2008) definition. In the condition of low normative conflict, participants received the message “The aforementioned policy is a good one and beneficial to the company because it helps to check whether each order is in line with the strategic objectives and avoid the harm of risky orders. In addition, most of your colleagues in the department agree that this policy is necessary”. In the condition of high normative conflict, participants received the message “In most cases, the aforementioned policy is a bad one and unbeneficial to the company because it reduces the efficiency of order processing and the quality of customer service. In addition, most of your colleagues in the department agree that this policy is unnecessary”.

### 4.3. Measurements

All the scales used to measure the variables were translated into Chinese using the translation and back-translation procedure. We measured organizational anomie with an eight-item scale developed by Choi et al. (2018). A sample item was “In our firm, the rules can be broken in order to achieve organizational goals” (1 = “strongly disagree”, 5 = “strongly agree”). Cronbach’s α for this scale was 0.804.

Manipulations were checked with four items. Two items (“Please evaluate the likelihood that Andy breaks the formal rules or prohibitions of the organization to help customers and colleagues”; “Please evaluate the likelihood that Andy breaks the formal rules or prohibitions of the organization to improve work efficiency”; 1 = “strongly unlikely” to 5 = “strongly likely”) were measured as MPSRB, and the other two items (“If you were the general manager of the company, you would reform the aforementioned policy”; “My company could be so much better if other policies could be used to replace the aforementioned policy”; 1 = “strongly disagree”, 5 = “strongly agree”) were measured as normative conflict.

Attention was checked with two items (i.e., “You are working in the sales department” and “Andy can push the order without approval from the Chief Customer Officer”; 0 = “yes”, 1 = “no”) to make sure participants think carefully about the material.

### 4.4. Results

#### 4.4.1. Manipulation Checks

An independent-sample *t*-test was used to check whether the manipulation of the variable was successful. The results showed that participants reported more MPSRB in the high-MPSRB condition than those in the low-MPSRB condition (t = 10.440, *p* < 0.01, Cohen’s d = 1.625), and reported more normative conflict in the high normative conflict condition than those in the low condition (t = 10.959, *p* < 0.01, Cohen’s d = 1.707). These results confirmed that the MPSRB and normative conflict manipulations were effective.

#### 4.4.2. Tests of the Hypotheses

In order to test the effect of MPSRB, normative conflict, and organizational anomie (Hypothesis 1 and Hypothesis 3), this study used organizational anomie as the dependent variable and conducted a 2 (high MPSRB vs. low MPSRB) × 2 (normative conflict: high vs. low) two-factor analysis of variance (ANOVA). The results showed that participants in the high-MPSRB condition (M = 3.030, SD = 0.472) reported higher organizational anomie than those in the low-MPSRB condition [M = 2.637, SD = 0.448; F (1, 163) = 30.135, η^2^ = 0.156, *p* < 0.01], indicating that Hypothesis 1 was supported. The results also showed that the effect of the interaction term between MPSRB and normative conflict on organizational anomie was significant [F (1, 161) = 13.520, *p* < 0.01, η^2^ = 0.077].

**Figure 2:**
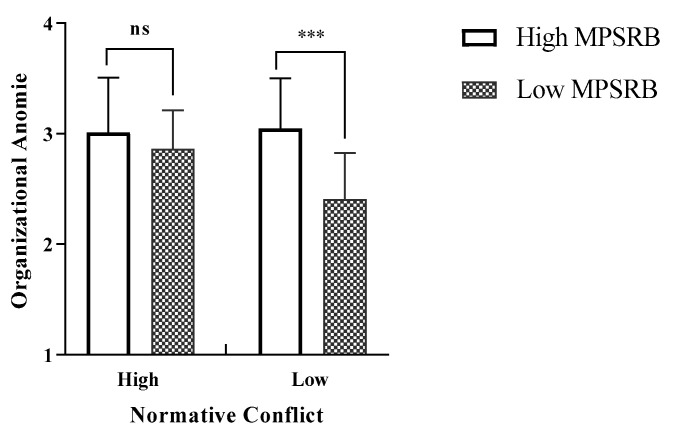
Interactive Effect of MPSRB and Normative Conflict on Organizational Anomie (Study 1). Notes. ns means nonsignificant; *** means *p* < 0.001. In the high MPSRB–high normative conflict condition, *n* = 42, in the high MPSRB–low normative conflict condition, *n* = 40; In the low MPSRB–high normative conflict condition, *n* = 42, in low MPSRB-low normative conflict condition, *n* = 41.

As depicted in Figure 2, in the low normative conflict condition, participants in the low-MPSRB condition reported a lower level of organizational anomie (M = 2.405, SD = 0.422) than participants in the high condition [M = 3.050, SD = 0.451; F (1, 161) = 44.893, η^2^ = 0.218, *p* < 0.01]. Conversely, in the high normative conflict condition, organizational anomie did not significantly differ between participants in the high-MPSRB condition (M = 3.012, SD = 0.497) and those in the low condition [M = 2.863, SD = 0.349; F (1, 161) = 2.482, η^2^ = 0.015, *p* > 0.05]. Thus, Hypothesis 3 was supported.

## 5. Study 2

### 5.1. Sample and Data Collection

Data were collected from a catering enterprise in China. We contacted HR or senior managers and explained the survey’s purpose to obtain permission. We ensured participant interest and voluntariness before the survey. This study collected data from supervisor–subordinate dyads. Considering the dual roles that some employees played in the organization, with the help of HR managers, we matched each employee with his supervisor one by one and ensured that the participant could only be “a subordinate” or “a supervisor”. We obtained dyad name lists from HR managers and assigned codes independently by researchers. Each questionnaire included the targeted participant’s code (for the first and third waves) and a confidentiality statement. When distributing questionnaires, researchers were verbally informed of the name represented by the code. Participants were asked to evaluate one direct supervisor or several subordinates without being informed of being evaluated. Finally, complete questionnaires were compiled based on the coded list.

Data were distributed and collected on-site in three waves to mitigate the potential for common method bias. In the first-wave survey (Time 1), we asked 450 employees to provide demographic information and complete the control variables, MPSRB of the direct supervisor, and normative conflict. In the second-wave survey (Time 2), which was conducted 30 days after Time 1, the employees reported their organizational anomie. In the third-wave survey (Time 3), which was conducted 30 days after Time 2, the employees’ immediate supervisors (including team leaders, supervisors, department heads, etc.) were asked to evaluate the workplace deviance of their subordinates. In the first wave of the survey, we received 383 valid employee questionnaires. In the second wave, we received 337 valid employee questionnaires. The effective response rate for the first two waves was 74.889%. In the third wave, questionnaires were distributed to the employees’ 83 supervisors. We acquired usable questionnaires from 74 supervisors (89.157% response rate). The resignation of nine supervisors resulted in the invalidation of 21 valid employee questionnaires. Finally, we obtained a sample of 316 valid supervisor–subordinate dyads questionnaires. During the survey, all enterprises operated smoothly and did not experience major upheavals. The main reason participants dropped out was their inability to be in the paper-and-pencil survey within the available time. The researchers did not observe their obvious negative emotions about the survey. This indicates that the quitting of some participants did not cause serious sample bias.

The demographic data of the subordinates showed that 159 respondents were males, and 157 were females. A total of 47.8% of the participants were between the ages of 31 and 40, and 45.3% of participants had worked in their organizations for 1–3 years. Considering positions, 79.7% were frontline employees, 16.8% were frontline managers, and 3.5% were middle-level managers, including project leaders, etc. For their superiors, the demographic variables showed that 75.7% were male, 48.6% of the superiors were between the ages of 41 and 50, and 56.8% of the superiors had worked in their organizations for 3–5 years.

### 5.2. Measures

The items were taken from existing scales which were considered reliable and valid. All items were translated from English to Chinese. This was performed with the parallel back-translation procedure to ensure the adequacy of meaning. Moreover, we consulted some employees in the enterprises and made several minor modifications to ensure the items could be generalized to the research context.

MPSRB: A 13-item scale developed by Dahling et al. (2012) was used to measure MPSRB. Since employees were asked to evaluate the PSRB of their direct supervisor in this study, we made two modifications. First, the instruction “Please evaluate whether your direct supervisor has the following behaviors. “ was added before the items. Second, the subject of the original items was changed from “I” to “he or she”. A sample item was “He or she assist other employees with their work by breaking organizational rules”. A five-point Likert scale was used for the measures (1 = never, 5 = very often). In this study, Cronbach’s α was 0.892 for efficiency, 0.883 for coworker assistance, 0.886 for customer service, and 0.928 for the overall scale.

Organizational anomie: As in Study 1, organizational anomie was measured by the eight-item scale developed by Choi et al. (2018). All items were rated on a five-point Likert scale ranging from 1 (strongly disagree) to 5 (strongly agree). Cronbach’s α for this scale was 0.890.

Employees’ workplace deviance: We used the scale developed by Bennett and Robinson (2000) to measure employees’ workplace deviance. It contains two dimensions, i.e., “interpersonal deviance” and “organizational deviance”. However, interpersonal workplace deviance often emerges in a hostile environment and develops into negative behaviors such as retaliating against other people; organizational workplace deviance is a negative behavior taken by employees against unfair workplace or unsatisfactory conditions of the organization (Gervasi et al., 2021). Thus, the “organizational deviance” subscale was more relevant to this study and used to measure the employees’ workplace deviance. The instruction “Please evaluate whether your immediate subordinate has the following behaviors. “was added before the items and we added the subject of the original items. A sample item included “He or she littered his or her work environment. “We rated all items on a five-point Likert scale ranging from 1 (never) to 5 (very often). Cronbach’s α for this scale was 0.910.

Normative conflict: The scale developed by Dahling and Gutworth (2017) was used to measure normative conflict. A sample item was “I think this organization will never reach its true potential until it changes its practices”. We also used a five-point Likert scale ranging from 1 (strongly disagree) to 5 (strongly agree) to rate all items. Cronbach’s α for this scale was 0.860.

Control variables: Previous studies have found that male, young, junior, and frontline employees are more likely to have deviance (Henle, 2005; Hollinger et al., 1992; Robinson & Greenberg, 1998). Therefore, we controlled for these demographic characteristics that might covary with the dependent variable. A 13-item scale developed by Yang and Diefendorff (2009) was used to measure managerial counterproductive work behavior directed at the organization. The instruction “Please evaluate whether your direct supervisor has the following behaviors. “was also added before the items. A sample item was “Left work earlier than you were allowed to” and we also changed it to “He or she left work earlier than he or she was allowed to. “A 5-point Likert scale was also used for rating all items (1 = never; 5 = very often). Cronbach’s α for this scale was 0.897.

### 5.3. Results

#### 5.3.1. Validity

**Figure 3:**
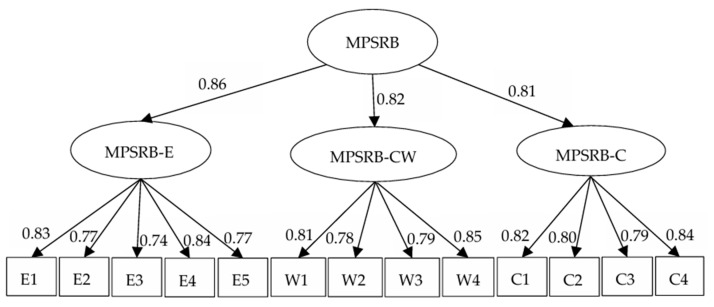
Confirmatory Factor Analysis of MPSRB. Notes: “MPSRB-E” means MPSRB for efficiency; “MPSRB-C” means MPSRB to help customers; “MPSRB-CW” means MPSRB to help co-workers.

Before testing the hypotheses, a Confirmatory Factor Analysis (CFA) using AMOS 26.0 was conducted to examine the discriminant validity of the related variables. First, we tested the factor structure of MPSRB and found that the three-factor model fit the data very well (χ^2^/df = 1.562, CFI = 0.986, TLI = 0.983, RMSEA = 0.042, IFI = 0.986), we also fixed all 13 PSRB items on one PSRB factor and the results showed the worse fit to the data (χ^2^/df = 9.547, CFI = 0.783, TLI = 0.740, RMSEA = 0.165, IFI = 0.784). The results also showed that the pairwise correlations among the three factors were high (between 0.660 and 0.700). Therefore, we modeled a higher-order MPSRB construct (shown in Figure 3), which was consistent with the study of Dahling et al. (2012). As a result, the higher-order factor model would be used in the following analysis. In addition, we also examined the discriminant validity of MPSRB, organizational anomie, normative conflict, employees’ workplace deviance, and managerial counterproductive work behavior. As shown in Table 1, the five-factor model fitted the data better (χ^2^/df = 1.416, RMSEA = 0.036, IFI = 0.930, TLI = 0.926, CFI = 0.930), which supported the discriminant validity of the six variables in this study.

#### 5.3.2. Descriptive Statistical Analysis

The means, standard deviations, and Pearson’s correlations are shown in Table 2. The results showed that MPSRB was positively correlated with organizational anomie (r = 0.390, *p* < 0.01), organizational anomie was positively correlated with the employees’ workplace deviant behavior (r = 0.212, *p* < 0.01), and managerial counterproductive work behavior was significantly related to MPSRB. Surprisingly, MPSRB was negatively correlated with employees’ workplace deviance (r = −0.219, *p* < 0.01), which was opposite to the mediating effect proposed, demonstrating that organizational anomie may not be only a mediator but also a suppressor variable in our research.

#### 5.3.3. Hypotheses Testing

To ensure that the hypothesis test results were not biased by multicollinearity, the variance inflation factor (VIF) tests were conducted and results showed that all VIF values were less than 1.5, indicating that there was no serious multicollinearity problem. We then tested the hypotheses with Hayes’ (2013) SPSS 26.0 software.

(1) Mediating Effect

The results are shown in Table 3. MPSRB showed a significantly positive relationship towards the organizational anomie (Model 2, b = 0.389, *p* < 0.001); thus, Hypothesis 1 was supported. Organizational anomie significantly predicted employees’ workplace deviance (Model 8, b = 0.297, *p* < 0.01). Furthermore, the results of the bootstrapping analysis indicated that the indirect effect of MPSRB on employees’ workplace deviance via organizational anomie was significant (indirect effect = 0.115, SE = 0.026, 95% CI [0.072, 0.177]). Consequently, Hypothesis 2 was supported. The Sobel test (Sobel, 1982) also confirmed this mediating effect (Z = 4.224, *p* < 0.001).

We also found that MPSRB negatively affected employees’ workplace deviance (Model 6, b = −0.273, *p* < 0.01), and the effect became bigger after entering the mediator (i.e., organizational anomie) (Model 8, b = −0.388, *p* < 0.01). It indicated that the influence of MPSRB on deviant behavior may contain other opposite mechanisms in addition to the broken windows effect, and we will try to explain it in the discussion.

(2) Moderating Effect

**Figure 4:**
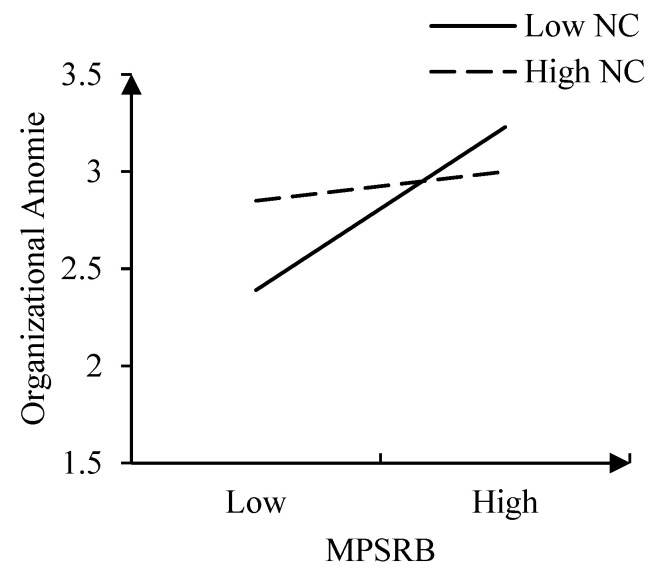
The Moderating Effect of Normative Conflict (Study 2).

The interaction term of MPSRB and normative conflict was included after being mean-centered (Aiken & West, 1991). The interaction had a significant negative effect on organizational anomie (Model 4, b = −0.424, *p* < 0.001). We then plotted the relationship between MPSRB and organizational anomie at one SD below and above the mean of normative conflict (Figure 4). Compared to high normative conflict (one SD above the mean) (effect = 0.138, se = 0.072, 95% CI [−0.004, 0.280], *p* > 0.05), MPSRB was significantly positively related to the organizational anomie (effect = 0.669, se = 0.075, 95% CI [0.521, 0.816], *p* < 0.01) when the normative conflict was low (1 SD below the mean). Thus, Hypothesis 3 was supported.

(3) Moderated Mediating Effect

We used the method proposed by Preacher et al. (2007) to test the moderated mediating effect by examining the conditional indirect effect at +1/−1 SD from the mean of normative conflict. The result of Table 4 indicated that when the normative conflict was one SD below the mean, the indirect effect was significant and stronger (effect size = 0.199, Boot SE = 0.040, 95% CI [0.124, 0.283]), while when the normative conflict was one SD above the mean, the indirect effect was non-significant and weaker (effect size = 0.041, Boot SE = 0.022, 95% CI [−0.002, 0.083]). In addition, the size of their difference reached −0.126 with a 95% confidence interval [−0.199, −0.070] excluding 0. Thus, Hypothesis 4 was supported.

(4) Supplementary Analyses

Following the suggestion of Becker et al. (2016), we excluded the key control variable (MCWB) and re-ran our model; the results shown in Table 5 once again proved the research hypotheses of this study (see also Confente & Kucharska, 2021). MPSRB still had a positive effect on organizational anomie (Model 10, b = 0.425, *p* < 0.001), which was larger than in Model 2 (b = 0.389, *p* < 0.001). The organizational anomie positively affected employees’ workplace deviance (Model 16, b = 0.338, *p* < 0.01), which was larger than in Model 8 (b = 0.297, *p* < 0.01). In addition, the total effect and indirect effect of MPSRB on employees’ workplace deviance through organizational anomie was more significant when the key control variables were included (total effect = −0.273, *p* < 0.01, 95% CI [−0.385, −0.160], with the key control variables; indirect effect = 0.115, *p* < 0.01, 95% CI [0.072, 0.177], with the key control variables; total effect = −0.219, *p* < 0.01, 95% CI [−0.333, −0.105], without the key control variables; indirect effect = 0.144, *p* < 0.01, 95% CI [0.092, 0.212], without the key control variables). Therefore, Hypothesis 5 was supported, controlling for MCWB could remove some meaningful variance in MPSRB and further enhance the validity of the research hypotheses.

## 6. Discussion

(1) Comparison between self-serving and altruistic rule-breaking behavior

This paper reveals that MPSRB exhibits a broken window effect, whereby it positively influences employees’ deviance via organizational anomie. The notion that witnessed rule-breaking, deviant, and unethical behavior will induce more negative behavior has been proven in various domains, such as crime and policing (Lanfear et al., 2020) and organizational management (Cialdini et al., 2021; Glomb & Liao, 2003; Robinson & O’Leary-Kelly, 1998). This effect is usually explained from several perspectives as follows: The first is selective attrition, stating that unethical leader behavior leads to the retention of group members more likely to engage in unethical behavior (Cialdini et al., 2021). The second is social learning, arguing that individuals engage in the same or similar deviant behavior by imitating others (Ewelt-Knauer et al., 2020). The third is social norms, pointing out that observing the unethical behavior of others can change the observer’s understanding of the descriptive social norms, making the observer feel less pressure to engage in this behavior (Gino et al., 2009). However, these viewpoints have their limitations. The view of selective attrition does not concern the behavior change in employees. The latter two views can only explain the contagion in the same deviant behavior. Although Lian et al. (2022) proposed a principle learning perspective to investigate the contagion across different deviance, the cognitive process behind it is towards a specific person, not the rule system itself. We need to know the change in one’s perception of rules after seeing others’ rule-breaking behavior. The research on the broken window effect in crime and policing may give us the answer from the point of view of the perceived rule failure (Lanfear et al., 2020).

Moreover, although these studies are regardless of whether the rule-breaking, deviant, and unethical behaviors are self-serving or altruistic, they are viewed negatively, at least by the participants. This leaves the question of whether altruistic rule-breaking, deviant, and unethical behaviors also have this dark side. Some research on ethical dilemmas has found that PSRB is morally problematic for formalists, who hold that “rule is rule” (Ishida et al., 2016; Reynolds & Ceranic, 2007). According to Kant’s interpretation of deontology or formalism, even out of altruistic motives, rule-breaking is un-universalized and harmful, reducing the effectiveness of rules (Offstein & Chory, 2017), which is consistent with institutionalism theory. As a result, we are inspired to transfer the broken windows effect to the organizational context to investigate MPSRB, which was confirmed in our study.

(2) MPSRB is a double-edged sword

Meanwhile, it was found that, in addition to the broken windows effect, MPSRB negatively and directly influences employees’ workplace deviance, providing evidence for a so-called suppression effect (Shrout & Bolger, 2002; Tzelgov & Henik, 1991). In other words, the mediator organizational anomie acts as a suppressor variable that buffers the negative direct effect. After including the suppressor (organizational anomie) and controlling for the noise of the broken windows effect, the negative direct effect of MPSRB becomes larger, demonstrating the bright side of MPSRB. As a result, MPSRB is a double-edged sword for employees’ workplace deviance. On the bright side, MPSRB is a proactive helping behavior (Morrison, 2006) that embodies a leader’s integrity, benevolence, and ethicality. As a result, it could initiate their positive reciprocal social exchange actions, such as reducing deviant workplace behavior, similar to servant leadership (Lapointe & Vandenberghe, 2018), empowering leadership (Lorinkova & Perry, 2017), etc. Furthermore, social learning, resource conservation, and emotion may also be useful for explaining this negative effect. This effect is interpersonal, social-emotional, and perceptible. On the dark side, MPSRB signals the failure of rules, which is impersonal, situational structural, hidden, and imperceptible, and therefore easy to ignore.

The competition between the bright and dark sides of MPSRB may depend on the social culture in which it is embedded. In national cultures that value results over process and substance over procedure, such as in China oriented toward pragmatic reasonability (Li, 2017), it is considered more reasonable and acceptable to break inhumane, cumbersome, and inefficient rules to improve the overall welfare. In such situations, the bright side of MPSRB dominates its dark side. The results of our study support this idea, that is, the total effect of MPSRB on deviant behavior is negative. In national cultures that value formalism over utilitarianism and universalism over particularism, such as in Western countries (Fassin, 2015), the reverse may be the case (Ishida et al., 2016; Reynolds & Ceranic, 2007).

(3) The influence of MPSRB and normative conflict on organizational anomie

We conclude that MPSRB positively affects the organizational anomie. Because MPSRB can reduce organizational standardization, which is negatively related to employees’ perception of organizational anomie. However, this relationship may be controversial. There are two contradictory views on the influence of organizational standardization on employees’ social psychological state. On the one hand, standardization is conducive to providing employees with clear work guidance and job responsibilities, decreasing role ambiguity (Podsakoff et al., 1986), reducing workplace disorder, and thus alleviating the sense of anomie (Swader, 2017). On the other hand, organizational standardization and work codification will cause employees to lack control over their work, and thus feel powerless and meaningless, which is an important manifestation of work anomie (Agarwal, 1993). The reason for this difference lies in the definition of anomie. The former effect is in line with the situation-structural definition, while the latter is in line with the social-emotional definition. Based on Durkheim’s ideas, organizational anomie is defined as lacking effective norms in our study. Therefore, MPSRB is positively related to organizational anomie. This is consistent with the prevailing view. For example, Teymoori et al. (2017) proposed two necessary conditions for anomie: disintegration and dis-regulation. The latter refers to illegitimate and ineffective leadership that undermines social regulation.

In Study 1, under high normative conflict conditions, employees’ organizational anomie is high, demonstrating that normative conflict has an additional positive effect on organizational anomie in addition to its moderating effect. This is consistent with previous studies, which argue that when informal rules and formal rules are imbalanced and conflict with each other, normative conflicts will be increased and organizational anomie will be formed (Adler, 2020). This suggests that normative conflict is a double-edged sword for organizational anomie, which is consistent with the research on the impact of normative conflict on some other variables, such as the perceived effectiveness of behavior (McDonald et al., 2013).

## 7. Practical Implications

The conclusions of this research can provide some enlightenment for management practices. First, organizations should restructure their rule managing activities to facilitate the creation of “good” rules and the abolition of “bad” rules, avoiding exposing managers to the dilemma of rule breaking, which will induce employees’ deviant behavior through organizational anomie. On the one hand, the rule making of the organization could follow the principles as follows: keep consistency among rules, allow for exceptions or flexibility, give priority to the needs of relevant groups, be simple rather than complex and time wasting, never convey distrust (Cropanzano & Byrne, 2001), etc., then the new rules may not conflict with the organization’s actual objectives in the future. On the other hand, existing rules should be regularly reviewed and checked. The red tape and outmoded policies should be identified and abolished in time, preventing the organization from the trap of rule overload.

Second, after engaging in pro-social rule breaking unavoidably, managers must take pertinent and effective measures promptly to compensate employees’ perception of organizational anomie caused by MPSRB. For example, they can timely and deeply communicate with their subordinates to explain the context, reasons, and consequences of the MPSRB or actively seek feedback from subordinates, focusing subordinates’ attention primarily on the “pro-social” rather than the “rule-breaking”. By performing so, managers may alleviate or eliminate subordinates’ doubts about the organizational norm and relieve the negative impact of MPSRB. In addition, organizations should struggle against employees’ perceived disintegration to minimize the sense of overall anomie in their daily work by shaping a tight organizational fabric with generalized interpersonal trust and consensual moral standards (Teymoori et al., 2017).

Finally, when facing the dilemma of whether or not to break the rules altruistically, managers should carefully consider the traits of organizational norms to bring them into harmony. In organizations where rule management is well performed, outdated rules have been updated in time, and the normative conflict is low, managers should be more prudent to stay away from PSRB and maintain the authority of the rule system as much as possible, which helps avoid the broken windows effect. However, in organizations where rule management is not emphasized enough, impractical rules are more tolerated, and the norm conflict is high, allowing managers to engage in pro-social rule breaking at their discretion to ensure substantial justice and organizational efficiency and strengthen the supervisor–subordinate guanxi through benevolence.

## 8. Limitations and Future Directions

Although the study has some contributions, there are also some limitations and directions for further research.

First, an additional finding revealed a negative and significant direct impact of MPSRB on employees’ workplace deviance, which was not explored in our study. This effect might stem from its “pro-social” rather than “rule-breaking” characteristic, suggesting that there could be other underlying mechanisms in the relationship between MPSRB and deviant behavior that remain unclear. In the future, we could investigate this effect from multiple perspectives, such as social exchange, social learning, and emotion, involving trust in the leader, ethical climate, work-related emotions, managerial trust, perceived organizational justice, other leadership styles and psychological empowerment, etc., as new moderators or mediators. Second, some conclusions may differentiate across different national cultures, such as the strength of the bright versus dark side of MPSRB. In our study, the data were collected from mainland China, where the national culture is characterized by “pragmatic reasonability”, in which the bright side of MPSRB dominates its broken windows effect. However, in other countries where formalism is preferred, the opposite may be true. Thus, future studies could collect data from multiple countries and across different cultures for cross-cultural research. Third, in our study, we measured organizational anomie following the view of Durkheim and defined it mainly as ineffectiveness and collapse of the organizational formal rules from the perspective of situational structure. That is part of the reason why MPSRB positively affects organizational anomie. However, there is another widespread definition of organizational anomie, i.e., the breakdown of attachment to an organization, which may result in different conclusions. Hence, future studies could develop a hyper-model to integrate the two types (maybe dimensions) of organizational anomie into the theory. Fourth, the impact of industry characteristics is lacking in our study. We should further study the broken window effect of MPSRB across different sectors and industries using qualitative and mixed methods and compare their differences.

## 9. Conclusions

Based on an experiment and a supervisor–subordinator paired questionnaires, the hypothesis proposed in this paper is verified. The conclusions are as follows: MPSRB will be like the broken window in organizations, where even small violations will have a cross-norm inhibition effect and enhance the organizational anomie. Based on goal-framing theory, when the organizational anomie is high, employees will increase organizational deviance to satisfy their interests. As a result, organizational anomie is the mediator through which MPSRB increases employees’ workplace deviance. Compared with high normative conflict, when the normative conflict is low, the rules violated by MPSRB are more authoritative, and MPSRB has a stronger broken windows effect, resulting in more perceived organizational anomie and more deviant behaviors. This paper has explored the dark side of MPSRB from the perspective of institutionalism. This aspect has been overlooked in previous research. By performing so, it deepens the comprehension of this form of constructive deviance and offers a novel perspective on the topic.

## Figures and Tables

**Table 1:** Confirmatory factor analysis results of all variables.

Models	χ^2^/df	RMSEA	IFI	TLI	CFI
Five-factor (MPSRB, MCWB, OA, NC, WDB)	1.416	0.036	0.930	0.926	0.930
Four-factor (MPSRB + MCWB, OA, NC, WDB)	1.714	0.048	0.880	0.874	0.879
Three-factor (MPSRB + MCWB + NC, OA, WDB)	2.322	0.065	0.777	0.766	0.776
Two-factor (MPSRB + MCWB + NC + OA, WDB)	2.996	0.080	0.663	0.646	0.661
Single -factor (MPSRB + MCWB + NC + OA + WDB)	3.945	0.097	0.502	0.478	0.499
Critical standards	<3	<0.05	>0.9	>0.9	>0.9

Notes: *n* = 316; MCWB = managerial counterproductive work behavior; WDB = employees’ workplace deviant behavior; NC = normative conflict; OA = organizational anomie.

**Table 2:** Means, standard deviations, and correlations of variables (Study 2).

Variables	Mean	SD.	1.	2.	3.	4.	5.	6.	7.	8.
1. Gender ^1^	0.500	0.501								
2. Age ^2^	2.300	0.722	−0.055							
3. Hierarchy ^3^	1.240	0.501	0.003	0.015						
4. Tenure ^4^	2.150	0.764	−0.047	0.121 *	−0.018					
5. MCWB	2.937	0.574	−0.015	0.015	0.014	0.038				
6. MPSRB	2.967	0.644	−0.067	0.058	0.018	0.027	0.194 **			
7. OA	2.871	0.691	−0.048	−0.072	−0.041	−0.026	0.237 **	0.390 **		
8. NC	2.519	0.624	−0.013	−0.013	0.088	0.054	−0.030	−0.027	0.113 *	
9. WDB	3.073	0.685	0.098	−0.079	−0.127 *	−0.137 *	0.201 **	−0.219 **	0.212 **	−0.053

Notes. *n* = 316. ** *p* < 0.01, * *p* < 0.05. ^1^ Gender was coded as follows: male = 1 and female = 0. ^2^ Age was coded as follows: <30 years = 1, 31–40 years = 2, 41–50 years = 3, and >51 years = 4. ^3^ Hierarchy was coded as follows: frontline employee = 1, frontline manager = 2, and middle-level manager = 3. ^4^ Tenure was coded as follows: <1 year = 1, 1–3 years = 2, 3–5 years = 3, and >5 years = 4.

**Table 3:** Hierarchical Regression Results (with MCWB).

Variables	Mediator (M): Organizational Anomie				Dependent (Y): Employees’ Workplace Deviance			
	Model 1	Model 2	Model 3	Model 4	Model 5	Model 6	Model 7	Model 8
Controls								
Gender	−0.068	−0.038	−0.066	−0.062	0.126	0.105	0.137	0.116
Age	−0.071	−0.088	−0.069	−0.093 *	−0.055	−0.043	−0.044	−0.017
Hierarchy	−0.059	−0.067	−0.075	−0.091	−0.179 *	−0.174 *	−0.170 *	−0.154 *
Tenure	−0.026	−0.030	−0.033	−0.038	−0.122 *	−0.120 *	−0.118 *	−0.111 *
MCWB	0.288 **	0.204 **	0.293 **	0.187 **	0.250 **	0.309 **	0.203 **	0.248 **
Independent								
MPSRB		0.389 ***		0.395 ***		−0.273 **		−0.388 **
Mediator								
OA							0.163 **	0.297 **
Moderator								
NC			0.139 *	0.111 *				
Interaction Term								
MPSRB × NC				−0.424 ***				
∆R^2^	0.067	0.126	0.015	0.208	0.091	0.063	0.025	0.135
∆F	4.457 **	48.090 **	5.215 *	29.388 **	6.215 **	22.874 **	8.839 **	26.946 **
Sobel test(Z)								4.224 ***
Indirect effect								0.115 **
Total effect						−0.273 **		
Direct effect						−0.388 **		

Notes. *n* = 316, *** *p* < 0.001, ** *p* < 0.01, * *p* < 0.05.

**Table 4:** Results of Moderated Mediating Effect.

Mediator	Conditions of Moderator	Mediating Effect	Boot SE	LL 95% CI	UL 95% CI
Organizational anomie	Low Normative Conflict	0.199	0.040	0.124	0.283
	High Normative Conflict	0.041	0.022	−0.002	0.083
	Difference	−0.126	0.033	−0.199	−0.070

Notes: *n* = 316; Bootstrap sample size = 5000; LL = lower limit; CI = confidence interval; UL = upper limit; High and Low level: Mean ± SD.

**Table 5:** Hierarchical regression results (without MCWB).

Variables	Mediator (M): Organizational Anomie				Dependent (Y): Employees’ Workplace Deviance			
	Model 9	Model 10	Model 11	Model 12	Model 13	Model 14	Model 15	Model 16
Controls								
Gender	−0.072	−0.038	−0.070	−0.064	0.123	0.105	0.137	0.118
Age	−0.069	−0.089	−0.067	−0.094 *	−0.053	−0.043	−0.039	−0.013
Hierarchy	−0.055	−0.064	−0.069	−0.088	−0.175 *	−0.170 *	−0.164 *	−0.149 *
Tenure	−0.018	−0.025	−0.024	−0.033	−0.115 *	−0.112 *	−0.112 *	−0.104 *
Independent								
MPSRB		0.425 ***		0.427 ***		−0.219 **		−0.363 **
Mediator								
OA							0.204 **	0.338 **
Moderator								
NC			0.130 *	0.105				
Interaction Term								
MPSRB × NC				−0.442 ***				
∆R^2^	0.010	0.155	0.014	0.242	0.047	0.042	0.042	0.139
∆F	0.771	57.602 **	4.293 *	33.270 **	3.848 **	14.337 **	14.263 **	26.409 **
Sobel test(Z)								4.715 ***
Indirect effect								0.144 **
Total effect						−0.219 **		
Direct effect						−0.363 **		

Notes. *n* = 316, *** *p* < 0.001, ** *p* < 0.01, * *p* < 0.05.

## Data Availability

All the datasets generated and analyzed during the current study are available from the corresponding author upon reasonable request.

## Appendix A

The experiment in Study 1 began with the following scenario description:

You work in the customer service department of a company that sells, leases, and services computer systems to small- and medium-sized businesses (e.g., PCs, servers, networking equipment, and printers). The responsibility of your department includes, among other things, taking orders from customers and processing them. Your immediate superior is Andy, the head of the department, who has been with the company for 3 years. Andy has just received a “hot order” from a large, important customer. This means that the order would have to be pushed to the front of the schedule and turned around within 24 h. Andy knows that there are strict policies in place for rush orders. One such policy is that the head of the customer service department is not allowed to rush such an order without approval from the Chief Customer Officer (CCO). Andy wants to be able to give the customer an answer because otherwise, they are likely to place the order with a competitor. But the CCO is tied up in meetings until the end of the day, so Andy cannot obtain the approval in time. Andy is considering whether to accept the order without approval, even though this would mean violating the policy, and he could reprimanded for this. Andy is really torn. Although he has nothing personally to gain by rushing this job, it would be good for the customer and might also be good for the company.
